# Exploratory assessment of serological tests to determine antibody titer against SARS‐CoV‐2: Appropriateness and limits

**DOI:** 10.1002/jcla.24363

**Published:** 2022-03-25

**Authors:** Alessandra Colombini, Marco Viganò, Rossella Tomaiuolo, Chiara Di Resta, Francesca Corea, Eleonora Sabetta, Davide Ferrari, Elena De Vecchi, Sestina Maria Spanò, Giuseppe Banfi

**Affiliations:** ^1^ 46767 Laboratorio di Biotecnologie Applicate all’Ortopedia IRCCS Istituto Ortopedico Galeazzi Milan Italy; ^2^ Vita‐Salute San Raffaele University Milan Italy; ^3^ 9372 IRCCS Ospedale San Raffaele Milan Italy; ^4^ 9372 SCVSA Department University of Parma Parma Italy; ^5^ 46767 Laboratory of Clinical Chemistry and Microbiology IRCCS Istituto Ortopedico Galeazzi Milan Italy; ^6^ 46767 IRCCS Istituto Ortopedico Galeazzi Milan Italy

**Keywords:** appropriateness, Luminex xMAP, multiplex assay, SARS‐CoV‐2, serological test

## Abstract

**Background:**

Serological tests can be used to detect antibodies in the serum of subject's after SARS‐CoV‐2 infection and vaccination. Currently, variability in antibody titers and the availability of a multiplicity of serological tests have made it necessary to highlight their appropriateness and limitations in various diagnostic settings.

**Methods:**

This study is part of Covidiagnostix, a multicenter project aimed at the assessment of the health technology used in SARS‐CoV‐2 serological tests. Based on data gained from the analysis of over 5000 subjects, a selected number of serum samples, representative of different diagnostic settings, were analyzed first by qualitative immunoassays (IgA, M, and G MILLIPLEX^®^ SARS‐CoV‐2 tests based on Luminex^®^) to define the immunoglobulins serum composition and subsequently by four serological diagnostic tests (Elecsys Anti‐SARS‐CoV‐2 and Elecsys Anti‐SARS‐CoV‐2 S by Roche, SARS‐CoV‐2 IgG by Siemens Healthcare, and CHORUS SARS‐CoV‐2 “NEUTRALIZING” Ab by DIESSE). The first WHO International Standard for SARS‐CoV‐2 was also analyzed using the same methods.

**Results:**

This study evaluated the antibody content and titer of the WHO Standard and serum of subjects with/without previous infection and before/after vaccination for SARS‐CoV‐2.

**Conclusion:**

The definition of antibodies in the WHO standard and the analysis of serum samples allowed for the identification of the appropriateness of serological tests in each diagnostic setting, increasing the effectiveness of the resulting laboratory data. Furthermore, we found that it would be optimal to produce new international standards against the S1 domain and RBD of the SARS‐CoV‐2 spike protein for a more effective serological monitoring of vaccination.

## Graphical abstract

Correlation plot represents the correlation between the result of the serological tests and MILLIPLEX^®^ SARS‐ CoV‐2 Antigen Panels. The strength of the correlation is represented by dot color and size, according to the continuous bar reported in the figure.

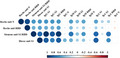

## 1 INTRODUCTION

The current gold standard for diagnosing severe acute respiratory syndrome coronavirus 2 (SARS‐CoV‐2) infection is “real‐time reverse transcription polymerase chain reaction (RT‐PCR),” which identifies the viral genome in samples taken from the respiratory tract and is particularly effective in the acute phase. On the other hand, serological tests allow for the detection of the presence of antibodies in the subject's serum from one to several weeks after infection or vaccination, which is the time necessary to produce antibodies. Serology is essential both for diagnosis, especially for patients with mild/moderate coronavirus disease 2019 (COVID‐19), who may present beyond the first 2 weeks of illness onset,^1^ as well as for the monitoring of the host immune response to viral antigen exposure.

It is essential to underline that the antibody titer varies between immunity due to natural infection and vaccination.^2^ In particular, after natural infection, the earliest developed antibodies are the secretory immunoglobulin A (IgA), which forms in the mucosal tissues of the nasal passages and gut, and the humoral immunoglobulin M (IgM). IgM are expressed on the plasma membrane of B cells and can be secreted in pentameric form. The binding of the IgM with the antigen determines the differentiation of the B cell into plasma cells to produce and secrete soluble antibodies with a high specificity for the antigen. Therefore, humoral immunoglobulin G (IgG) forms later than IgM but is characterized by a higher specificity and guarantees a longer term protection than IgM. Long‐lasting protection is ensured by B cells that differentiate into memory B cells. In the event of a new encounter with the same antigen, they differentiate into plasma cells to rapidly produce high specificity IgG. The efficacy of vaccines that guarantee long‐term protection and the production of specific IgG through the involvement of memory B cells is based on this mechanism.^3^

Circulating IgA antibodies appear 4–24 days after infection, appearing after 11 days in most cases. The levels of IgM antibodies are detectable from 4 to 14 days after infection and increase until about the 20th day (peaking between 2 and 5 weeks), after which they begin to disappear, declining over 3–5 weeks post‐symptom onset. The IgG antibodies become detectable 12–15 days from infection, that is, at a later time compared to the IgM, with a peak between 3 and 7 weeks and the ability to persist for at least 8 weeks.^4^

Considering the antigen specificity of the immunoglobulins, upon viral infection, the humoral immune system responds by producing antibodies against multiple SARS‐CoV‐2 proteins, including the spike (S) and the nucleocapsid (N) protein. The spike (S) proteins form the characteristic “corona,” or crown, of the virus and are composed of subunit S1, which contains the receptor‐binding domain (RBD), and subunit S2, containing the fusion peptide. The spikes surround the membrane glycoprotein and the envelope protein, containing the viral RNA encased by the N protein.^5^ Upon vaccination, the humoral immune system is able to potentially develop antibodies against spike proteins but not against N proteins.^6^

All IgA, IgM, and IgG can be measured in blood serum and plasma samples.^4^ In vitro serological tests detecting the presence of specific antibodies are used to reveal past infections and vaccine reactivity. By testing the response of each type of immunoglobulin against specific antigenic regions of SARS‐CoV‐2, it is also possible to track the immune response to the virus during COVID‐19 infection and recovery. Therefore, these tests are essential for epidemiological assessments of population seroprevalence and forward‐looking estimates of global therapeutic needs.

To date, numerous antibody serological tests are on the market in traditional, automated, and point‐of‐care forms; various manufacturing companies have developed commercial kits that exploit different immunological assays. The so‐called rapid tests that detect IgM and IgG in capillary or venous blood samples through immuno‐chromatographic methods are purely qualitative and therefore exclusively indicate the presence or absence of antibodies without their precise quantification. ELISA (enzyme‐linked immunosorbent assay), CLIA (automated chemiluminescent immunoassay), or ECLIA (electrochemiluminescent immunoassay) tests, on the other hand, are diagnostic tests that identify IgA, IgM, IgG, and total Ig in serum or plasma samples with a generally high sensitivity and specificity. Although their sensitivity is very low in the first week after the onset of symptoms, this improves in the second week and reaches its maximum at 21 days after infection^7^ or vaccination.

However, many of these commercially available serologic tests have been recalled due to their poor performance. In fact, the majority have not been fully evaluated with large panels of samples, stressing the importance of their systematic validation or a health technology assessment (HTA) approach.^8^

To maximize the informativeness of the serological tests, there is a need to specify the antibodies that they determine and at which limits. As the antibody responses reflect exposure to virus and vaccination, to ensure the appropriateness of the request, the effectiveness, and the correct interpretation/communication of the laboratory data, it is essential to assess serological tests in different diagnostic settings.

Aiming to improve the effectiveness of laboratory data, this study focuses on the definition of the most appropriate serological test to be used in the different diagnostic settings (patient seropositive to SARS‐CoV‐2 or vaccinated subjects). To reach this objective, the main goal of the present study is to perform a qualitative analysis of the antibodies (class and antigen recognition) present in the sera of specific groups of patients (with and without previous SARS‐CoV‐2 infection and before/after vaccination) and in the WHO standard was performed.

## 2 MATERIALS AND METHODS

### 2.1 Study design

This study is part of *Covidiagnostix*, a multicenter national project granted by the Italian Ministry of Health aimed to evaluate several diagnostic serological tests available on the market for SARS‐CoV‐2 using a health technology assessment (HTA) approach.^8^ Five Italian Scientific Institutes for Research, Hospitalization and Healthcare (IRCCS) and one Cooperative for home care took part in the *Covidiagnostix* project: IRCCS Ospedale San Raffaele Hospital (OSR) and IRCCS Orthopedic Institute Galeazzi (IOG) (Milano, Italy); IRCCS Casa Sollievo della Sofferenza (CSS) (San Giovanni Rotondo, FG, Italy); Fondazione IRCCS Policlinico San Matteo (Pavia, Italy); IRCCS Ospedale Pediatrico Bambino Gesù; and OSA Cooperative a r. l. (Roma, Italy). The participants are involved in the COVID‐19 epidemic in terms of both assistance and scientific research, providing a network of skills ranging from laboratory testing and analytical evaluation to the management of processes related to serological tests.^8^, ^9^, ^10^, ^11^, ^12^

Based on experience gained from the qualitative and semi‐quantitative analysis of over 5000 subjects, the first WHO International Standard and a small number of serum samples representative of all the analyzed patient groups were further tested using a bead‐based multiplex assay to define the serum composition (class and antigen recognition) of immunoglobulins against SARS‐CoV‐2.

### 2.2 Serum samples

The samples were selected in the context of different diagnostic settings:

- Nine serum samples obtained from subjects infected by the virus and not vaccinated, denoted as “natural seropositive,” were sampled 20 days after the onset of symptoms.
- Five serum samples obtained from subjects who recovered from a previous SARS‐CoV‐2 infection and then received one dose of the BNT162b2 mRNA COVID‐19 vaccine, denoted as “vaccinated natural seropositive”; they were sampled 21 days after the vaccination.
- Twenty serum samples obtained from subjects who received the BNT162b2 mRNA COVID‐19 vaccine without a previous SARS‐CoV‐2 infection, denoted as “vaccinated seronegative”; since the seropositive samples received only the first vaccine dose, seronegative subjects were collected at the same time point, 21 days after the first dose.

As a negative sample, four serum samples obtained in 2018 before the SARS‐CoV‐2 pandemic, denoted as “pre‐pandemic,” were used.

Written informed consent was collected according to the Ethical Review Board of IRCCS San Raffaele Hospital (protocol no. CE:199/INT/2020; date of approval December 23rd, 2020, approved by the IRCCS San Raffaele Hospital Ethical Review Board).

In addition, the first WHO International Standard for anti‐SARS‐CoV‐2 human immunoglobulin (NIBSC code: 20/136) was tested. It represents a pooled plasma obtained over 28 days after the onset of symptoms from 11 individuals who recovered from SARS‐CoV‐2 infection.^13^

### 2.3 Methods

#### 2.3.1 Multiplex assays

The three different MILLIPLEX^®^ SARS‐CoV‐2 tests (Millipore Sigma), a bead‐based multiplex assay based on Luminex^®^ xMAP^®^ technology, were used to detect the presence of IgA, IgG, and IgM against SARS‐CoV‐2 spike protein subunits S1 and S2, RBD and N in the 38 samples and the first WHO international standard.

Briefly, MILLIPLEX^®^ SARS‐90 CoV‐2 Antigen Panel 1 IgA (ref: HC19SERA1‐85K), IgG (ref: HC19SERG1‐85K), and IgM (ref: HC19SERM1‐85K) were used to analyze the serum samples in duplicate. This assay format requires capture beads conjugated with specific SARS‐CoV‐2 antigens to be incubated with 1:100 dilution of human serum samples to form bead‐analyte sandwiches, subsequently detected by adding anti‐human immunoglobulin type‐specific antibodies conjugated to the phycoerythrin fluorophore (PE). The fluorescent signal emitted by each bead with its associated bound immunoassay sandwich is finally read on the Luminex^®^ MAGPIX™ Instrument System. These qualitative assays provide the results as median fluorescent intensity (MFI) and do not include standards for quantitation; therefore, running non‐infected control samples is required to establish an experimental MFI cutoff. In fact, pre‐pandemic sera were used to define the cutoff for the presence of the antibodies of interest in the different samples (Table 1).

**TABLE 1:** Threshold positivity, sensitivity, and specificity of the MILLIPLEX^®^ SARS‐CoV‐2 tests

Test	Antibody	Antigens	Threshold (MFI)	Sensitivity(%)	Specificity(%)
MILLIPLEX^®^ SARS‐CoV−2 Panel 1 IgA	IgA	S1	54	100	100
		S2	484	100	100
		RBD	440	90	100
		N	522	50	75
MILLIPLEX^®^ SARS‐CoV−2 Panel 1 IgM	IgM	S1	201	90	100
		S2	325	100	75
		RBD	1293	80	100
		N	721	100	100
MILLIPLEX^®^ SARS‐CoV−2 Panel 1 IgG	IgG	S1	1069	100	100
		S2	7677	100	100
		RBD	2836	100	100
		N	854	100	100

Intra‐assay precision results for all three panels were declared to be <15% CV, calculated from the mean of the %CVs from eight reportable results in a single assay. Inter‐assay precision for all three panels was <20% CV, calculated from the mean of the %CVs across four different assays.

#### 2.3.2 Diagnostic serological tests

The serum samples and the first WHO International standard were also tested to determine the antibody titer against SARS‐CoV‐2 antigens, using:

- The Elecsys Anti‐SARS‐CoV‐2 (Roche Diagnostics, Inc.), a high‐throughput ECLIA qualitative method to detect Pan‐Ig against the Nucleocapsid protein.
- The Elecsys Anti‐SARS‐CoV‐2 S (Roche Diagnostics, Inc.), a high‐throughput ECLIA qualitative and semi‐quantitative method to detect Pan‐Ig against the spike RBD.
- The SARS‐CoV‐2 IgG (sCOVG) (Siemens Healthcare Diagnostics Inc.), a high‐throughput CLIA qualitative and semi‐quantitative approach to detect IgG against the spike RBD.
- The CHORUS SARS‐CoV‐2 “NEUTRALIZING” Ab (DIESSE Diagnostica Senese SpA), an immunoenzymatic method for the quantitative detection of Pan‐Ig against the spike (S1) protein.

The characteristics of the diagnostic serological tests are listed in Table 2.

**TABLE 2:** Characteristics of evaluated commercial serological test for the detection of anti‐SARS‐CoV‐2 antibodies

Test	Developer	Technology	Target	Antibody	Sensitivity (%) (PPA)	Specificity (%) (NPA)	Cutoff	Manufacturer's datasheet
Elecsys Anti‐SARS‐CoV−2	Roche Diagnostic Inc.	High‐throughput ECLIA (qualitative)	Nucleocapsid	Pan‐Ig	100 95% CI: 88.3%–100%	99.8 95% CI: 99.7%–99.9%	1.0	ref: 09203079190
Elecsys Anti‐SARS‐CoV−2 S	Roche Diagnostics Inc.	High‐throughput ECLIA (qualitative and semi‐quantitative)	Spike (RBD)	Pan‐Ig	96.6 95% CI: 93.4%–98.3%	100 95% CI: 99.9%–100%	<0.80 U/ml: negative From ≥0.80 U/ml to ≤250 U/ml: positive >250: positive	ref: 09289275190
SARS‐CoV−2 IgG (sCOVG)	Siemens Healthcare Diagnostics Inc.	High‐throughput CLIA (qualitative and semi‐quantitative)	Spike (S1 RBD)	IgG	95.6 95% CI: 92.3%–97.5%	99.9 95% CI: 99.6%–100%	1.00–100 Index	ref: 11207490_EN rev. 02
CHORUS SARS‐CoV−2 NEUTRALIZING Ab	DIESSE	Immunoenzymatic method (quantitative)	Spike (S1)	Pan‐Ig	99.6 95% CI: 97.7%–99.9%	99.8 95% CI: 99.2%–99.9%	>50.0 BAU/ml: positive <20.0 BAU/ml: negative 20.0‒50.0 BAU/ml: equivocal	ref: 81408

### 2.4 Statistical analysis

Receiver‐operating characteristic was used to set putative thresholds in a multiplex assay to identify positive and negative samples. The pre‐pandemic and natural seropositive samples were used to establish experimental cutoffs. Thresholds for positivity were determined maximizing Youden's index (Table 1).

Kolmogorov‐Smirnov test was used to assess the normality of the data distribution, and unpaired ANOVA or Kruskal‐Wallis tests were used to compare multiplex assay data among natural seropositive, seronegative, or seropositive vaccinated subjects.

One‐vs‐one Spearman's correlation coefficients were calculated between the values obtained from each method and the experimental values (MFI) for IgA, IgM, and IgG, using data from seronegative vaccinated subjects. These numerical results are reported in the text (Spearman's coefficient and *p*‐value) and graphically in the figures using a correlation plot where the size and color of dots represent the magnitude and the sign of the correlation coefficient.

Antibody titers demonstrated a beta distribution determined by comparing theoretical and empirical distribution functions. Then, beta regression models (using R package “betareg”) were used to assess the contribution of the different antibody classes against the different antigens to determine the test results.

## 3 RESULTS

**FIGURE 1:**
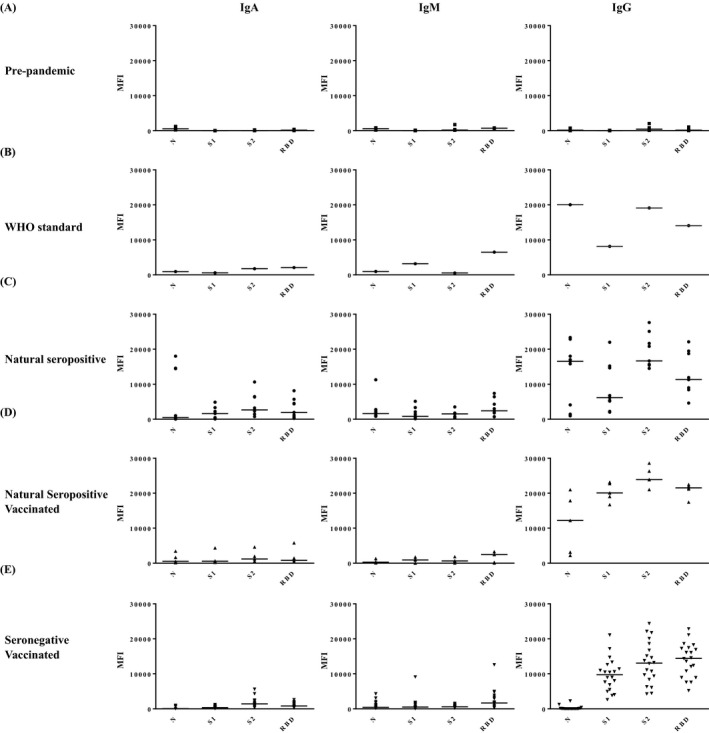
Graph representing the results of the different assayed groups of samples run in MILLIPLEX^®^ SARS‐CoV‐2 Antigen Panel 1 IgA, IgM, and IgG 4‐plex analytes: N, S1, S2, RBD. Dot plots show individual dots for each sample's median fluorescent intensities (MFIs). Line indicates the median values

The serum samples and the first WHO international standard were simultaneously tested by the MILLIPLEX^®^ SARS‐CoV‐2 antigen panels for IgA, IgM, and IgG antibodies against SARS‐CoV‐2 S1 S2, RBD, and N antigenic proteins, as reported in Figure 1 (panels A‐E).

The first WHO International standard sample (Figure 1B) shows MFI levels above the determined threshold for all the antigenic proteins of IgA, IgM, and IgG, proving that all these antibodies are present in the standard. The MFI levels defined in the natural seropositive were similar to the WHO standard (Figure 1C).

The natural seropositive vaccinated subjects (Figure 1D) showed MFI levels above the IgA, IgM, and IgG threshold against S1, S2, and RBD antigens.

The seronegative vaccinated subjects (Figure 1E) show MFI levels above the IgA, IgM, and IgG threshold against S1, S2, and RBD antigens.

Both seronegative and seropositive subjects were negative for IgM against N antigen. Vaccinated seronegative subjects were also negative for IgA and IgG against N antigen.

Table 3 summarizes the positivity and negativity for IgA, IgM, and IgG against different epitopes in natural seropositive patients and vaccinated subjects. Thresholds obtained from the ROC curves (Table 1) were used as internal references to define the results shown in Table 3.

**TABLE 3:** Positivity and negativity for IgA, IgM, and IgG against S1, S2, RBD, and N in natural seropositive, natural seropositive vaccinated, and seronegative vaccinated subjects obtained by multiplex assays

	IgA				IgM				IgG			
	S1	S2	RBD	N	S1	S2	RBD	N	S1	S2	RBD	N
Natural seropositive	+	+	±	±	±	+	±	+	+	+	+	+
Natural seropositive vaccinated	+	+	±	±	±	+	±	−^##^	++^*,##^	++^***^	++^*,#^	+^**^
Seronegative vaccinated	+	+^#^	±	−	±	±^#^	+/‐	−^##^	+	±^#^	+	−^###^

Note

In the presence of some samples below the positivity threshold, this was indicated as ±.

*p < 0.05, **p < 0.01, ***p < 0.001 vs. seronegative vaccinated.

^#^ *p* < 0.05, ^##^ *p* < 0.01, ^###^ *p* < 0.001 vs.natural seropositive.

In addition to the differences concerning the presence/absence of antibodies against N antigen, the comparison of seronegative vaccinated subjects with natural seropositive showed significantly lower levels (*p* < 0.05) of all the three immunoglobulins against S2 antigen in seronegative vaccinated. On the contrary, higher IgG levels against S1 and RBD antigens were observed in seropositive vaccinated subjects compared to natural seropositive subjects. The comparison of data obtained from seronegative and seropositive vaccinated subjects showed that those that were vaccinated seropositive had higher levels of IgG against S1 (*p* < 0.05), S2 (*p* < 0.001), and RBD (*p* < 0.05) compared with vaccinated seronegative subjects.

**FIGURE 2:**
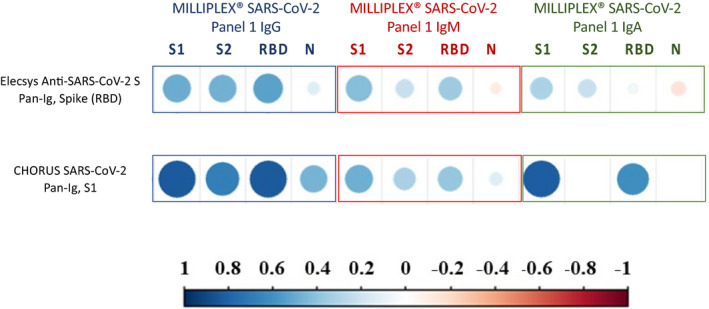
Correlation plot representing the correlation between the result of the Pan‐Ig serological test and MILLIPLEX^®^ SARS‐ CoV‐2 Antigen Panel 1 IgG (bordered in blue), IgM (bordered in red), and IgG (bordered in green). The strength of the correlation is represented by dot color and size, according to the continuous reference bar reported in the figure

Correlations between the data obtained from MILLIPLEX^®^ SARS‐CoV‐2 antigen panels for IgA, IgM, and IgG and Pan‐Ig diagnostic serological tests are shown in Figure 2. The Elecsys Anti‐SARS‐CoV‐2 S test (detecting Pan‐Ig anti‐RBD) results moderately correlate with MILLIPLEX^®^ SARS‐CoV‐2 antigen panels for IgG only, regardless of the recognized antigen on the spike protein S1 (*r* = 0.525, *p* = 0.018), S2 (*r* = 0.490, *p* = 0.028), or RBD (*r* = 0.571, *p* = 0.008); no significant correlation was observed for the N antigen.

The other Pan‐Ig test, CHORUS SARS‐CoV‐2 “NEUTRALIZING” Ab, showed a strong correlation with IgG anti‐S1 (*r* = 0.835, *p* < 0.001), IgG anti‐RBD (*r* = 0.863, *p* < 0.001), and IgA anti‐S1 (*r* = 0.821, *p* < 0.001), as well as moderate correlation with IgG anti‐S2 (*r* = 0.683, *p* = 0.001), IgM anti‐S1 (*r* = 0.484, *p* = 0.031), and IgA anti‐RBD (*r* = 0.611, *p* = 0.004).

**FIGURE 3:**
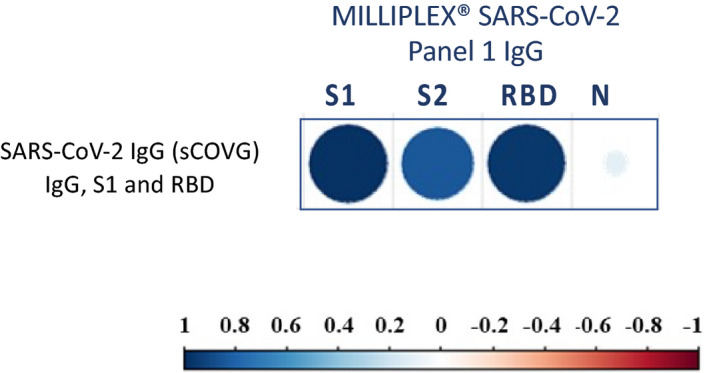
Correlation plot representing the correlation between the result of the SARS‐CoV‐2 IgG (sCOVG) test and MILLIPLEX^®^ SARS‐ CoV‐2 Antigen Panel 1 IgG (bordered in blue). The strength of the correlation is represented by dot color and size, according to the continuous reference bar reported in the figure

The correlation between the results of SARS‐CoV‐2 IgG (sCOVG) and MILLIPLEX^®^ SARS‐CoV‐2 antigen panels for IgG was very strong for anti‐S1 (*r* = 0.981, *p* < 0.001), anti‐S2 (*r* = 0.841, *p* < 0.001), and anti‐RBD (*r* = 0.964, *p* < 0.001; Figure 3).

Using multiple beta regression models to assess the antibodies contributing the most to the results of each specific test, it emerged that the Elecsys Anti‐SARS‐CoV‐2 S test was significantly influenced by IgG against RBD only (*p* < 0.0001), the CHORUS SARS‐CoV‐2 “NEUTRALIZING” Ab test results depended substantially on all three antibody class anti‐S1 (IgG: *p* < 0.0001, IgA: *p* = 0.004, IgM: *p* < 0.0001), and the results of the SARS‐CoV‐2 IgG (sCOVG) depended mainly on anti‐S1 IgG (*p* < 0.0001) rather than IgG anti‐RBD (*p* = 0.0001).

**FIGURE 4:**
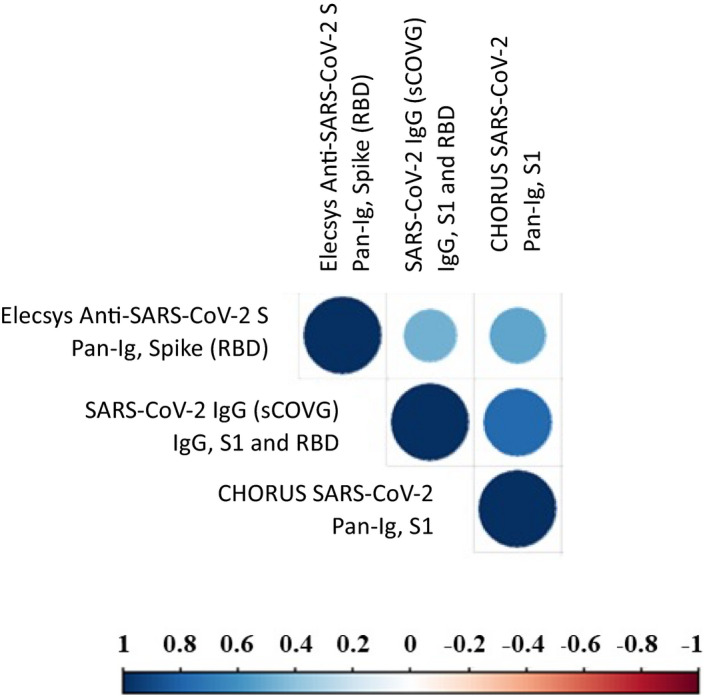
Correlation plot representing the correlation between the diagnostic serological tests. The strength of the correlation is represented by dot color and size, according to the continuous reference bar reported in the figure

Considering the correlation between the tests for antibodies against S1 and RBD antigens, Elecsys Anti‐SARS‐CoV‐2 S (detecting Pan‐Ig anti‐RBD) was moderately correlated with SARS‐CoV‐2 IgG (sCOVG) (detecting IgG anti‐S1/RBD; *r* = 0.505, *p* = 0.023) and CHORUS SARS‐CoV‐2 “NEUTRALIZING” Ab (detecting Pan‐Ig anti‐S1; *r* = 0.583, *p* = 0.007). Instead, a stronger correlation was observed between SARS‐CoV‐2 IgG (sCOVG; detecting IgG anti‐S1/RBD) and CHORUS SARS‐CoV‐2 “NEUTRALIZING” Ab (detecting Pan‐Ig anti‐S1; *r* = 0.771, *p* < 0.001; Figure 4).

Finally, the only test against N antigen was Elecsys Anti‐SARS‐CoV‐2; consequently, the serological results obtained did not correlate with any other diagnostic serological test.

## 4 DISCUSSION

This study provides for the first time representative expected antibody profile (class and antigen recognition) of the serum of subjects with and without previous infection by SARS‐CoV‐2 and before/after vaccination with BNT162b2. Further information concerning the composition of the WHO standard in terms of immunoglobulins against specific antigens was also reported and is fundamental to harmonize the results obtained from the wide range of inherently different assays available.

The main findings of the study confirmed that IgG against N antibodies are the best choice to identify subjects naturally exposed to SARS‐CoV‐2, while also indicating and suggested that IgG against S1, S2, and RBD should be used to monitor the antibody response to the vaccine.

In addition, an exploratory evaluation of the consistency in the titer determined by four available diagnostic laboratory tests with the specific antibody content is provided. The determination of antibodies directed against the SARS‐CoV‐2 nucleocapsid protein and spike protein (e.g., S1/S2 domains or the RBD receptor's binding domain) is widely used to characterize host antibody responses to SARS‐CoV‐2 infection or vaccination. The qualitative and quantitative analysis of antibodies against SARS‐CoV‐2 of the same 38 samples carried out in this study allowed us to evaluate the appropriateness of the diagnostic request in the three most frequent diagnostic contexts: subjects who contracted the virus and were not subsequently vaccinated (natural seropositive), subjects who recovered from COVID‐19 and then vaccinated (natural seropositive vaccinated), and subjects who received the vaccine without previously having contracted the virus (seronegative vaccinated).

To assess the appropriateness of the request, serological tests were divided into those that recognize the nucleocapsid protein and those that recognize the spike protein or its components.

The request of serological tests against N protein (e.g., Elecsys Anti‐SARS‐CoV‐2), testing the antibody response developed to SARS‐CoV‐2 infection, is appropriate to determine the antibody titer in subjects who contracted the virus both symptomatically and asymptomatically. The serological test can be beneficial for asymptomatic subjects. The presence of the N antigen allows the identification of those subjects who have contracted the virus, even if they have not developed the disease. If they never contracted the virus, both vaccinated and unvaccinated subjects will be negative for the N antigen. In particular, non‐vaccinated subjects who have never contracted the virus are negative for any antigen.

The natural seropositive samples strictly relate to WHO standards for all antibody classes and antigens, suggesting that these samples are representative of the expected antibody content for subjects who were infected by SARS‐CoV‐2. Therefore, the N antibody test identifies these natural seropositive individuals compared to uninfected subjects, whether vaccinated or not.^7^, ^14^, ^15^

In subjects who received the BNT162b2 mRNA COVID‐19 vaccine without having previously contracted the virus, these tests give a negative response due to the absence of antibodies against the N protein. However, a positive result indicates a SARS‐CoV‐2 infection after vaccination: in this case, the test may be requested to identify a post‐vaccination infection. Conversely, requesting the test in subjects who have recovered from COVID‐19 and been vaccinated with the BNT162b2 mRNA COVID‐19 vaccine is not valid as a test of the vaccine's efficacy, as the antibody titer against N protein refers to natural infection and not only to vaccination. The same is applicable to other vaccines based on mRNA or DNA to produce Spike proteins (e.g., AZD1222 or CX‐024414). On the contrary, detecting the anti‐N is essential to distinguish between vaccinated seronegative and natural seropositive subjects.

The request for serological tests against S protein, testing the antibody response mounted after the SARS‐CoV‐2 infection and the vaccine, is appropriate to determine the antibody titer both in subjects who have contracted the virus and were not subsequently vaccinated and in vaccinated subjects with or without having previously contracted the virus. While there is currently no direct evidence of a correlation between antibody titer and protection from SARS‐CoV‐2 infection or symptoms, the presence of antibodies could be considered an indicator of an immune response to the vaccine.^16^

In our samples, IgG against S1, S2, and RBD were present in natural seropositive, vaccinated seropositive, and vaccinated seronegative subjects, with IgG against S1 and RBD highly present in vaccinated seropositive in comparison with the other two groups and IgG against S2 highly present in both natural and vaccinated seropositive in comparison with vaccinated seronegative subjects. The differences among the antibody profiles of these specific subgroups of subjects could be exploited to design more sensitive assays to better monitor the antibody response in vaccinated seropositive and seronegative subjects.

A lower level of IgA was observed in vaccinated subjects (seropositive and seronegative) compared to natural seropositive; this is possibly due to the relationship between IgA response and the localization of infection in the mucosa,^17^ which is absent in seronegative vaccinated individuals and relatively distant in time for seropositive vaccinated subjects. In fact, systemic immunity does not necessarily translate into mucosal immunity. This has important implications when providing intramuscular injections for a virus that enters via the upper respiratory tract and adds to growing evidence that a vaccination that elicits a mucosal response is required.^18^, ^19^, ^20^, ^21^

The available diagnostic laboratory tests for the definition of the antibody titer present several differences in terms of both recognized antigen and antibody class. However, among the analyzed tests that target the S protein (Elecsys Anti‐SARS‐CoV‐2 S by Roche; SARS‐CoV‐2 IgG (sCOVG) by Siemens, and CHORUS SARS‐CoV‐2 “NEUTRALIZING” Ab by Diesse), a particularly strong correlation was observed between the serological tests by Siemens and Diesse; these two tests were set up to recognize IgG and all classes of immunoglobulins, respectively, against S1 antigen (and RBD in the case of Siemens).

On the other hand, the Elecsys Anti‐SARS‐CoV‐2 S by Roche specifically recognizes RBD antigen (all Ig classes) and showed only a strict relationship with IgG. The lack of S1 recognition may explain the observed reduced correlation with the other anti‐S1 tests. Nevertheless, the Roche test would be less sensitive to variations in IgA and IgM titer occurring during time compared to the other Pan‐Ig test by Diesse.

The SARS‐CoV‐2 IgG (sCOVG) results by Siemens against S1 and RBD antigens appeared to depend strongly on anti‐S1 antibodies rather than anti‐RBD.

According to its design, the Diesse test effectively identifies all anti‐S1 antibody classes. Based on these observations, assays based on IgG against S1 antigens (or eventually designed exploiting IgG against RBD) should be selected to monitor the immune response after the vaccine.

The small sample size represents the main limitation of the study; thus, these preliminary data will need to be verified on a larger cohort of subjects. Other limitations are related to the fact that the current data represent a reference only for subjects undergoing vaccines based on mRNA or DNA to produce S proteins, and should be considered as strictly related to the time‐point explored after vaccination and to the seronegative vaccinated subjects in terms of serological observations.

## 5 CONCLUSION

Serological analysis is capable of defining the antibody profile in classes of immunoglobulins and antigens against SARS‐CoV‐2 in natural seropositive, vaccinated natural seropositive, and vaccinated seronegative subjects and the WHO standard. As expected, the natural seropositive samples strictly relate to the WHO standard for all antibody classes; moreover, the most appropriate tests to identify individuals naturally exposed to SARS‐CoV‐2 are those based on recognizing IgG against N antibodies.

Due to the relevant presence of IgG against S1, S2, and RBD in natural seropositive, vaccinated seropositive, and vaccinated seronegative subjects, with differences among groups and to the observed correlation among serological results, tests exploiting IgG against S1 or RBD antigens should be selected to monitor the immune response after vaccination.

This study highlights the need to produce new international standards against the S1 and RBD domains of the SARS‐CoV‐2 spike protein, preferably on an industrial scale, allowing for the use of serology to monitor vaccination reactivity more effectively.

## CONFLICT OF INTERESTS

The authors declare that they have no conflict of interest.

## AUTHOR CONTRIBUTIONS

Conceptualization: AC, RT, CR, and GB; formal analysis: AC and MV; investigation: AC, RT, CR, FC, ES, DF, EV, and SMS; methodology: AC, DF, EV, and SMS; supervision: AC, RT, CR, and GB; writing – review & editing: AC, MV, FC, ES, EV, and SMS; writing – original draft: RT and CR; data curation: FC, ES, DF, EV, and SMS; funding acquisition: GB.

## INSTITUTIONAL REVIEW BOARD STATEMENT

The study was conducted according to the guidelines of the Declaration of Helsinki and approved by the Institutional Review Board (CE: 199/INT/2020).

## PATIENT CONSENT STATEMENT

Informed consent was obtained from all subjects involved in the study.

## Data Availability

The data presented in this study are available on request from the corresponding author.
